# Minimally invasive versus open Transforaminal lumbar Interbody fusion in obese patients: a meta-analysis

**DOI:** 10.1186/s12891-018-1937-6

**Published:** 2018-01-17

**Authors:** Qingsong Xie, Jing Zhang, Feng Lu, Hao Wu, Zan Chen, Fengzeng Jian

**Affiliations:** 1 Department of Neurosurgery, Xuanwu Hospital, Capital Medical University, Division of Spine, China International Neurological Institute, Beijing, People’s Republic of China; 2 Department of Neurosurgery, Wenzhou Medical University Affiliated Cixi Hospital, Cixi, Zhejiang, People’s Republic of China; 3 Department of Neurosurgery, Capital Medical University Affiliated Beijing Friendship Hospital, Beijing, People’s Republic of China; 4 Department of Neurosurgery, south branch of Fujian provincial Hospital, Fuzhou, People’s Republic of China

**Keywords:** Transforaminal lumbar interbody fusion, Obese, Lumbar degenerative diseases, Meta-analysis

## Abstract

**Background:**

Minimally invasive transforaminal lumbar interbody fusion (MI-TLIF) has been employed in increasing cases compared with open TLIF (Open-TLIF). However, it is uncertain whether the advantages of MI-TLIF can also be specifically applied in obese patients. Therefore, the current study was thereby carried out aiming to compare the outcomes of MI-TLIF with those of Open-TLIF in obese patients with lumbar degenerative diseases.

**Methods:**

Electronic databases were systemically retrieved from construction to May 2017. Meanwhile, the odds ratio (OR), mean difference (MD) and 95% confidence intervals (CI) were determined.

**Results:**

A total of 7 observational cohort studies were enrolled into the current meta-analysis. The results indicated that, compared with Open-TLIF group, MI-TLIF could remarkably reduce the operative time (*P* = 0.002), intraoperative blood loss (*P* < 0.001), postoperative drainage (*P* = 0.01), length of stay (P < 0.001) and incidence of complications (P < 0.001). In addition, MI-TLIF could also lead to markedly lower early back pain-Visual Analog Scale (BP-VAS) score than that of Open-TLIF (P < 0.001), but no statistically significant differences were found in Oswestry Disability Index (ODI), late BP-VAS, early leg pain-VAS (LP-VAS) and late LP-VAS scores.

**Conclusion:**

MI-TLIF may be a more preferred choice for obese patients undergoing spinal surgery. However, differences in the long-term functional and pain outcomes between MI-TLIF and Open-TLIF remain a source of controversy, which should be further verified in future randomized-control trials.

**Electronic supplementary material:**

The online version of this article (10.1186/s12891-018-1937-6) contains supplementary material, which is available to authorized users.

## Background

The economic development and changes in people’s work and lifestyle have rendered obesity an independent risk factor of low back pain (LBP), which has become the health care crisis worldwide [1]. According to the National Institutes of Health [2], the obese patients are those with a body mass index (BMI) of over 30 without significant comorbidity. Strikingly, the prevalence of severe obesity has been steadily rising; therefore, the proper surgical management for the severely obese population remains an increasingly important issue.

Currently, spine surgeons are encountered with a new challenge in managing the obese ([BMI] > 30) and morbidly obese (BMI > 35) patients undergoing lumbar spinal fusion surgery, which can be attributed to the poor operative corridors and difficult access to necessary anatomical landmarks [3, 4]. Specifically, obese patients have posed unique technical operative challenges due to the increased complexity and greater complications compared with those in nonobese patients, which may thus result in different association between operative approach and clinical outcomes [5–7]. However, traditional open transforaminal lumbar interbody fusion procedure (Open-TLIF) will result in greater damage to muscle and soft tissue, in the meantime of adding to blood loss and the risk of infection in obese patients with lumbar disc herniation, since it frequently requires extensive line of incision [8, 9]. Fortunately, the minimally invasive transforaminal lumbar interbody fusion (MI-TLIF) technique has emerged within the last decade. MI-TLIF is superior to Open-TLIF in its less postoperative pain, less intraoperative blood loss, and shorter length of stay [10, 11].

Systematic evidences have investigated the efficacy of spinal fusion [12–14], laminectomy [15], discectomy [16], and pedicle screw fixation [17] between MI-TLIF and Open-TLIF. However, to the best of our knowledge, no review has analyzed the perioperative, functional, and pain outcomes between MI-TLIF and Open-TLIF in obese population. Consequently, it remains unclear whether MI-TLIF or open-TLIF procedure will result in superior postoperative functional outcomes in treating obese population with degenerative lumbar diseases. Therefore, the current study was thereby carried out aiming to explore which surgical technique was more beneficial for obese patients.

## Methods

### Retrieval strategy

Electronic databases, including Pubmed, Web of Science, the Cochrane database, China National Knowledge Internet (CNKI) and the Wanfang Database, were systemically retrieved from construction to May 2017 using the following terms, transforaminal lumbar interbody fusion, minimally invasive, TLIF, minimally invasive spine surgery, obesity, obese, body mass index, BMI. and spinal fusion. Specifically, only English-language or Chinese-language citations were taken into account. All pooled analyses were independently conducted by two investigators, and any disagreement was settled by mutual discussion. A flowchart illustrating information identification, screening, eligibility, and the finally enrolled studies was constructed according to *Preferred Reporting Items for Systematic Reviews and Meta-analyses* (PRISMA) guidelines [18]. The current systematic review was not registered, and no protocol was available. Moreover, the meta-analysis was checked using the terms presented in the PRISMA list (Additional file 1: Table S1).

### Selection criteria

The study inclusion criteria were as follows: (i) study with the minimum sample size in each group of 10; (ii) study including a comparative design (MI-TLIF versus open-TLIF); (iii) studies mentioning at least one of the following outcomes: operative time, blood loss, postoperative drainage, length of stay, complications, and pre- and postoperative functional and pain scores assessed by Oswestry Disability Index (ODI) and visual analog scale (VAS); (iv) study enrolling the population of adult patients classified as obesity; and (v) comparative study (randomized controlled trial (RCT), cohorts, case-controls and observational studies). Specifically, obesity was defined as a BMI of > 30 kg/m2 [19]. Exclusion criteria were as follows: (i) review articles, editorial comments, meta-analyses; duplicated studies and guidelines, (ii) study with the sample size in each group of less than 10, and (iii) study with no placebo agent control group.

### Data extraction

Data were extracted by two reviewers independently. Any disagreement between the two reviewers in data extraction was settled by the opinion of a third reviewer. Briefly, the following information was extracted from the trials: study design, patient demographics, performed interventions, outcomes of interest, statistical methods, and study results. Moreover, for dichotomous outcomes, the number of participants experiencing the outcome and the number assessed in each treatment group were recorded.

### Study outcomes

In the current meta-analysis, the primary outcomes were mean improvements in back and/or leg pain Visual Analog Scale (VAS) scores, and mean improvement in Oswestry Disability Index (ODI) score. Outcomes were categorized into early (≤6 months after surgery) and late (≥ 1 year after surgery) [12] depending on the above 2 primary outcomes at the end of follow-up. In addition, secondary outcomes include operative time, intraoperative blood loss, postoperative drainage, length of stay (LOS), and number of complications.

### Quality assessment

Two authors had independently assessed the quality of each trial to evaluate the risk of bias in the included studies. Meanwhile, the quality of nonrandomized studies was evaluated using the Newcastle–Ottawa Scale (NOS), discriminating between case-control trials and cohort studies [20]. NOS is a scale recommended by the Cochrane Non-Randomized Studies Methods Working Group. NOS will address 3 areas when analyzing case-control trials, including selection, comparability and exposure. In comparison, it will deal with selection, comparability and outcome in cohort studies. Specifically, a quality score of 0–9 points is allocated to each nonrandomized study, and those achieving ≥7 points are considered to be of high quality. Notably, such scale had been developed for application in systematic reviews and meta-analyses.

### Statistical analysis

Dichotomous and continuous variables were analyzed using odds ratios (ORs) and mean differences (MDs) [21]. Meanwhile, inter-study heterogeneity was assessed using Cochran’s Q-statistic test and heterogeneity between the studies included was evaluated using chi-square test, with a *P* < 0.05 indicating significant heterogeneity. The random effects model would be employed in the presence of heterogeneity between studies, which would provide a more conservative effect than the fixed-effects model [22]. In addition, sensitivity analysis would also be performed in the case of heterogeneity by eliminating one study at a time, so as to check for the resolution of heterogeneity. Besides, the publication bias was assessed using the visual funnel plot [23]. Data were analyzed using the Review Manager (RevMan version 5.3; Cochrane Collaboration, Oxford, UK).

## Results

### Study selection

**Fig. 1:**
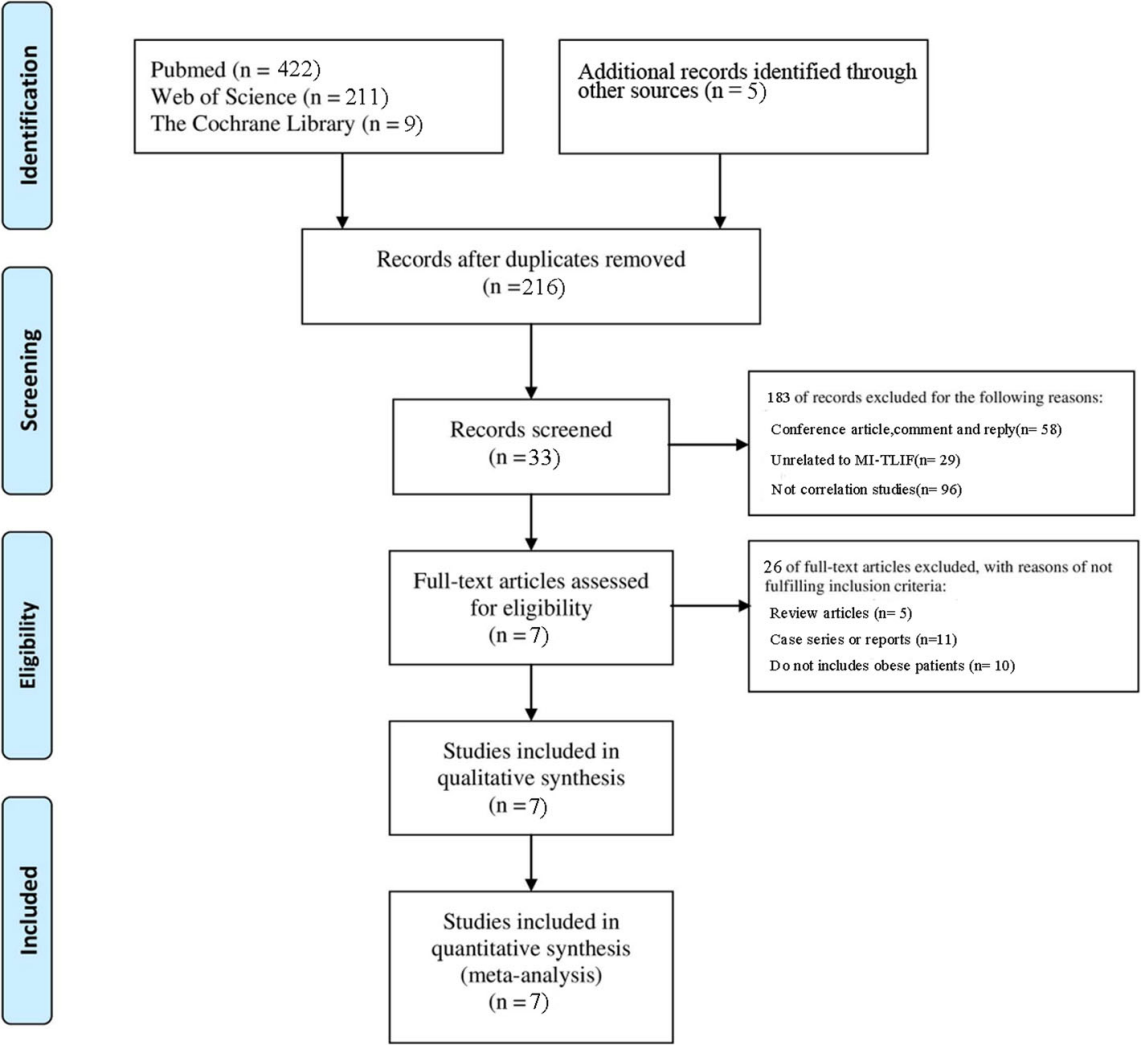
Study selection flow diagram for the meta-analysis

A total of 647 potential trials were identified in the initial retrieval strategy, among which, 431 duplicates were eliminated. Meanwhile, some additional studies were excluded based on the inclusion criteria. Meanwhile, altogether 33 citations were retrieved for detailed evaluation of the full text, 26 of which were excluded due to their nature of case series and review articles or without the involvement of obese patients. Finally, 7 observational studies were identified in the final analysis [24–30]. All studies were identified and the number of studies subsequently included or excluded was illustrated as a flow chart (Fig. 1).

### Characteristics of trials

One out of the 7 identified studies was prospective comparative study, whereas the remaining 6 were retrospective comparative studies. A total of 638 patients were enrolled in the identified observational studies, which were published between 2013 and 2017. The NOS was employed to evaluate the quality of nonrandomized studies, among which, a majority were considered to be of moderate quality. The detailed information of the enrolled studies was presented in Tables 1 and 2.

**Table 1:** Characteristics of studies included in the meta-analyses

Study	Study	No. of patients	Mean follow	Meanage	Gender (% male)	Mean BMI (kg/m2)	Diagnosis	NOS score
	design	(MI: Open)	up (mo)	(y) (MI: Open)	(MI: Open)	(MI: Open)		
Adogwa [24], USA	retrospective cohort study	40/108	24	56.62/56.12	50/47	34.48/35.63	DDD, Spondylolisthesis	7
Wang [29], China	retrospective cohort study	35/37	6	51.3/52.3	54/68	34.8/33.7	LDP	6
Lau [25], USA	retrospective cohort study	78/49	NP	50.5/57.4	46.2/42.1	36.9/37.2	spondylolisthesis, DDD, LDH, stenosis, deformity	7
Wang [28], China	prospective cohort study	42/39	36.1	56.4/54.2	69.1/69.2	29.5/28.3	spondylolisthesis,	6
Terman [27], USA	retrospective cohort study	53/21	30	52.4/58.2	45/62	35.2/33.8	spondylolisthesis, DDD, stenosis, LDH	7
Zhang [30], China	retrospective cohort study	32/24	6	42/45	41/39	31.3/33.2	LDH	5
Mao [26], China	retrospective cohort study	46/33	6	40.8/43.3	41.3/36.3	32.8/33.6	LDH	5

*DDD* Degenerative disc disease, *LDH* Lumbar disc herniation, *NOS* Newcastle Ottawa Scale, *MI* Minimally invasive surgery, *Open* Open surgery, *NP* Not provided, *mo* Month, *y* Year

**Table 2:** Summary of MI-TLIF and O-TLIF Studies Eligible for Analysis

Study	Complication(s)		Measures of Functional and Pain Outcomes		Operative Time (min),Blood Loss(ml), LOS (d),and Postoperative drainage(ml)	
	MI-TLIF	Open-TLIF	MI-TLIF	Open-TLIF	MI-TLIF	Open-TLIF
Adogwa [24], USA	5(2 surgical-site infection; 1 Spinal cord/nerve root injury; 1 Durotomy;1 Hardware failure)	12(1 surgical-site infection; 1 Spinal cord/nerve root injury; 9 Durotomy;1 adjacent segment disease)	BP-VAS(1 year,2 year) = 2.62 ± 3.82,2.42 ± 3.81; LP-VAS(1 year,2 year) = 3.35 ± 4.77,3.77 ± 4.53; ODI(1 year,2 year) = 17.09 ± 26.73,11.61 ± 25.52	BP-VAS(1 year,2 year) = 3.50 ± 3.70,2.33 ± 3.67; LP-VAS(1 year,2 year) = 3.03 ± 4.34,2.67 ± 4.10; ODI(1 year,2 year) = 18.43 ± 22.41,14.88 ± 22.1	NP	NP
Wang [16], China	0	3(2 fat liquefaction;1 infection)	BP-VAS(3mo,6mo) = 1.6 ± 0.9,1.0 ± 0.4; ODI(3mo,6mo) = 19.9 ± 3.0,17.1 ± 2.3	BP-VAS(3mo,6mo) = 2.4 ± 1.2,1.8 ± 0.5; ODI(3mo,6mo) = 20.8 ± 1.0,16.5 ± 2.2	Time = 152 ± 56; BL = 136 ± 18; LOS = 4.7 ± 1.2; PD = 52 ± 10	Time = 103 ± 31; BL = 364 ± 23; LOS = 8.6 ± 3.1; PD = 375 ± 26
Zhang [30], China	2fat liquefaction	2fat liquefaction	BP-VAS(5d) = 2.11 ± 1.25; LP-VAS(5d) = 1.86 ± 1.11; ODI (5d) = 15.9 ± 1.23	BP-VAS(5d) = 2.8 ± 1.6; LP-VAS(5d) = 2.3 ± 1.9; ODI (5d) = 2.4 ± 1.1	Time = 118 ± 26; BL = 126 ± 49; LOS = 6 ± 2.7	Time = 188 ± 41; BL = 430 ± 76; LOS = 10 ± 4.2
Mao [26], China	3fat liquefaction	3(1dural laceration,2fat liquefaction)	BP-VAS(5d,3mo,6mo) = 2.09 ± 1.23, 1.39 ± 0.23, 0.39 ± 0.13; LP-VAS(5d,3mo,6mo) = 1.78 ± 1.03,1.09 ± 1.03, 0.46 ± 0.21; ODI(5d,3mo,6mo) = 27.3 ± 3.01, 15.9 ± 1.23, 7.2 ± 0.98	BP-VAS(5d,3mo,6mo) = 2.6 ± 1.40, 1.78 ± 0.33, 1.09 ± 0.13; LP-VAS(5d,3mo,6mo) = 2.3 ± 1.90,1.79 ± 0.23, 0.89 ± 0.12; ODI(5d,3mo,6mo) = 30.2 ± 2.01, 18.2 ± 2.21, 12.2 ± 0.92	Time = 120 ± 28.26; BL = 110.83 ± 50.51; LOS = 5 ± 2.5	Time = 200 ± 43.05; BL = 420 ± 86; LOS = 9.3 ± 3.4
Wang [28], China	4(2 Superficial wound infection 2 Dural tear)	7(4 Superficial wound infection 3 Dural tear)	BP-VAS (1 day,30mo) = 1.5 ± 0.7, 1.3 ± 0.6; ODI (30mo) = 18.2 ± 5.9	BP-VAS (1 day,30mo) = 3.8 ± 1.4, 1.3 ± 0.6; ODI (30mo) = 17.4 ± 7.1	Time = 127 ± 25; BL = 274 ± 99; PD = 52 ± 23	Time = 168 ± 37; BL = 645 ± 163; PD = 190 ± 84
Terman [27], USA	9(1 cardiopulmonary; 2 durotomy; 1 K-wire fracture; 2 urinary tract infection; 1pneumonia;1 ileus; 1 urinary retention)	11(3durotomy; 5 excessive blood loss; 1 seroma; 1 wound infection;1 urinary retention)	BP-VAS (30mo) = 2.4 ± 2.35; ODI (30mo) = 15 ± 23.3	BP-VAS (30mo) = 2.8 ± 2.087;ODI (30mo) = 13 ± 21.969	Time = 100 ± 25; LOS = 2 + 0.5;	Time = 550 ± 175;LOS = 3.25 + 0.25;
Lau [25], USA	9(2 durotomy 1 fractured K-wire in L-5 vertebral body 1 wound dehiscence atrial fbrillation w/ rapid ventricular 1response 2 UTI(urinary tract infection) 1 tachycardia associated w/ respiratory failure 1 deep vein thrombosis)	14(8 durotomy 1valium w/drawal 1development of seroma 1reoperation for screw revision 1 UTI(urinary tract infection) 1 tachycardia associated w/ respiratory failure; 1 wound infection)	NP	NP	BL = 168.6 ± 162.1; LOS = 3.1 ± 1.7;	BL = 661.0 ± 561.3; LOS = 4.7 ± 2.1

*BP-VAS* Back pain-visual analog scale, *LP-VAS* Leg pain-visual analog scale, *ODI* Oswestry disability index, *BL* Blood Loss, *LOS* Length of stay, *PD* Postoperative drainage, *MI-TLIF* Minimally invasive transforaminal lumbar interbody fusion surgery, *Open-TLIF* Open transforaminal lumbar interbody fusion surgery, *NP* Not provided

### Visual analog scale (VAS)

**Fig. 2:**
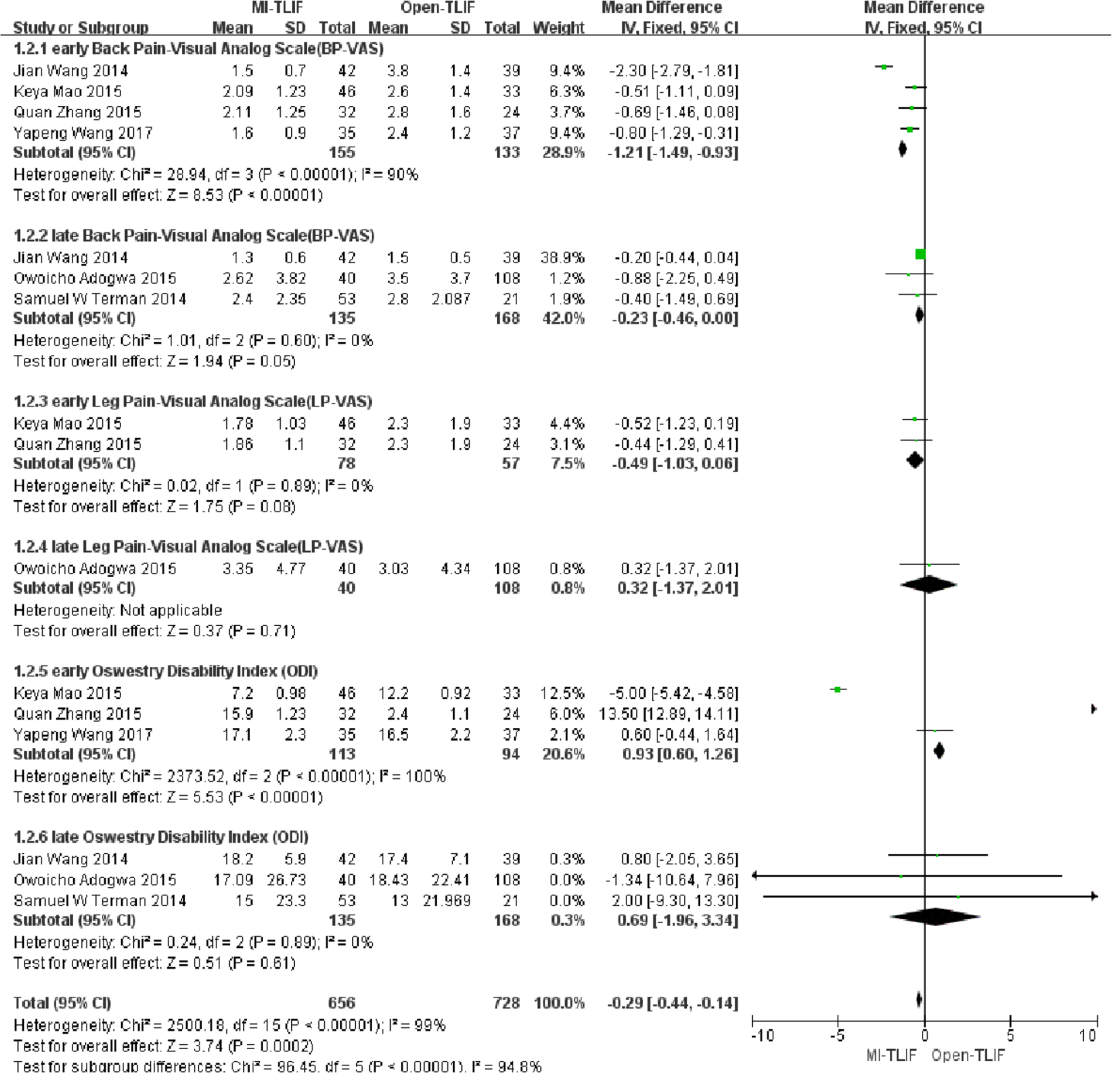
Forest plots comparing final pain outcomes between minimally invasive and open spinal fusion treatments with (1) early back pain-visual analog scale (BP-VAS), (2) late BP-VAS, (3)early leg pain-visual analog scale(LP-VAS) and (4) late LP-VAS

Altogether 4 studies [26, 28–30] harbored sufficient data about the early back pain-visual analog scale (BP-VAS) scores (≤6 months after surgery) and 3 [24, 27, 28] mentioned sufficient data regarding the late BP-VAS scores(≥ 1 year after surgery). Moreover, 2 studies [26, 30] covered enough data on the early leg pain-visual analog scale (LP-VAS) scores (≤6 months after surgery) and 1 [24] on the late LP-VAS scores (≥ 1 year after surgery). Meanwhile, no differences were founded in late BP-VAS, early LP-VAS or late LP-VAS scores between two groups. However, significant differences were found in early BP-VAS (MD = − 1.09; 95%CI = − 1.98, − 0.21; *p* = 0.02) between MI-TLIF and Open-TLIF groups. Furthermore, significant heterogeneity was detected among the studies only in the early BP-VAS group (I2 = 90%, *P* < 0.001). (Fig. 2).

#### Oswestry disability index

**Fig. 3:**
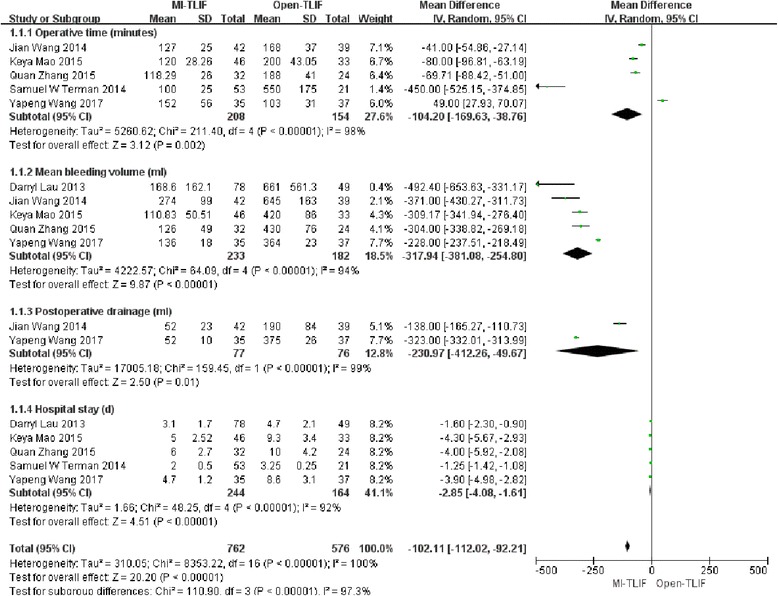
Forest plots comparing final functional outcomes between minimally invasive and open spinal fusion treatments with (1) early oswestry disability index (ODI) and (2) late ODI

In total, 3 studies [26, 29, 30] covered sufficient data on the early ODI scores(≤6 months after surgery) and 3 [24, 27, 28] on the late ODI scores(≥ 1 year after surgery). No differences were founded in early ODI or late ODI. At the same time, significant heterogeneity was observed among the studies only in the early ODI group (I2 = 100%, *P* < 0.001). (Fig. 3).

#### Operative time

**Fig. 4:**
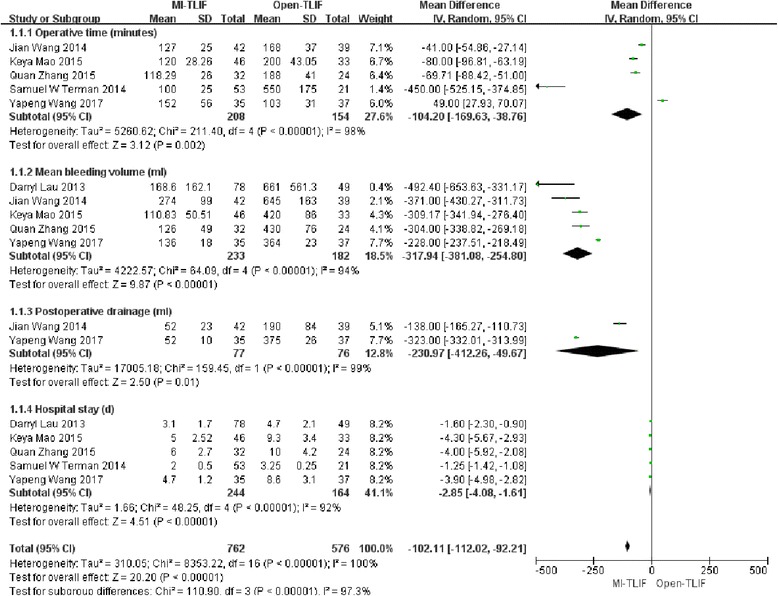
Forest plots comparing perioperative outcomes between minimally invasive and open spinal fusion treatments for (1) operative time (minutes), (2) intraoperative blood loss (mL), (3) postoperative drainage and (4) length of stay (days)

In total, 5 studies [26–30] mentioned enough information on the estimated operative time. The pooled results indicated that patients undergoing MI-TLIF had less operative time (MD = − 104.2; 95%CI = − 169.63, − 38.76; *p* = 0.002), and the difference was statistically significant. Meanwhile, significant heterogeneity was also observed among the studies (I2 = 98%, *P* < 0.001). (Fig. 4).

#### Intraoperative blood loss

Five studies [25, 26, 28–30] covered enough information on the estimated intraoperative blood loss. The pooled results demonstrated that patients receiving MI-TLIF had less intraoperative blood loss (MD = − 317.97; 95%CI = − 381.08, − 254.80; *p* < 0.001), with the difference being statistically significant. In the meantime, significant heterogeneity was detected among the studies (I2 = 94%, *P* < 0.001). (Fig. 4).

#### Postoperative drainage

Two studies [28, 29] had sufficient data on the estimated postoperative drainage. The pooled results suggested that patients experiencing MI-TLIF had less postoperative drainage (MD = − 230.97; 95%CI = − 412.26, − 49.67; *p* < 0.001), and the difference was statistically significant. Also, significant heterogeneity could be observed among the studies (I2 = 99%, *P* < 0.001). (Fig. 4).

#### Length of stay (LOS)

Five studies [25–27, 29, 30] reported the LOS. The pooled results indicated that patients receiving MI-TLIF had shorter LOS (MD = − 2.85; 95%CI = − 4.08, − 1.61; *p* < 0.001), and the difference was statistically significant. Significant heterogeneity was also detectable among the studies (I2 = 92%, *P* < 0.001). (Fig. 4).

### Complications

**Fig. 5:**
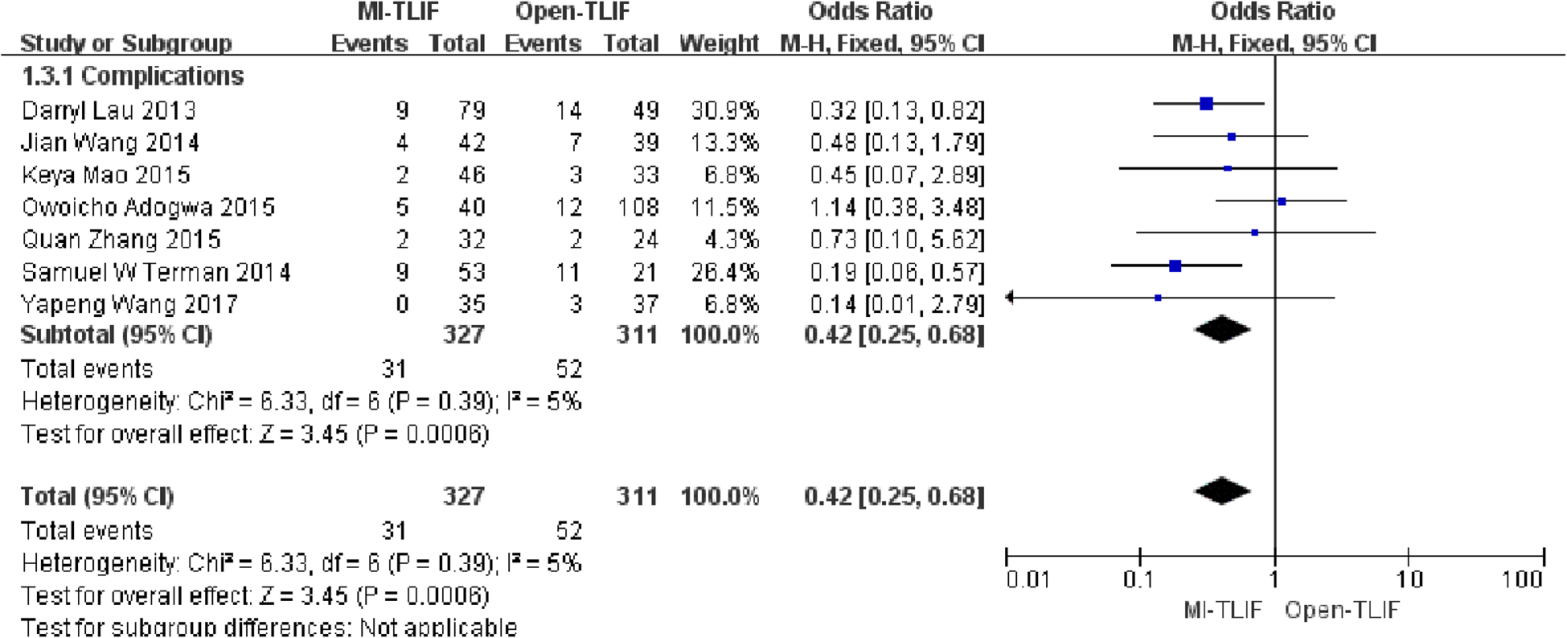
Forest plot comparing complications between minimally invasive and open spinal fusion treatment

All the 7 trials had reported the incidence of complications in the MI-TLIF group and Open-TLIF group of 9.5% (31/327) and 16.7% (52/311), respectively. Notably, patients undergoing MI-TLIF had markedly lower rates of complications (OR 0.42; 95% CI 0.25–0.68; *p* < 0.001). There was no heterogeneity among the selected studies evaluating the clinical treatment (I^2^ = 5%, *P* = 0.39). (Fig. 5).

### Sensitivity analysis and publication bias

**Fig. 6:**
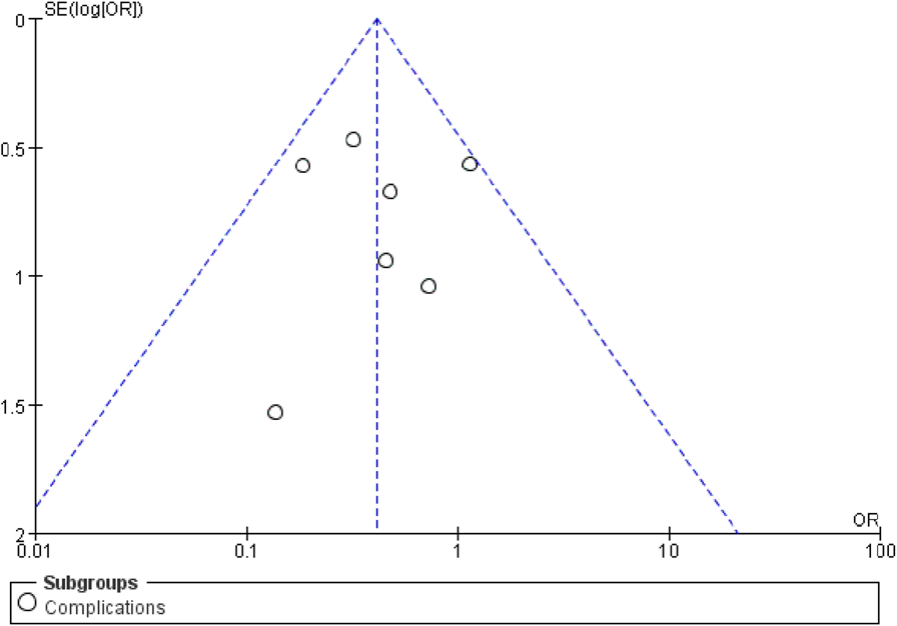
Funnel plot to detect publication bias. No significant funnel asymmetry that could indicate publication bias was observed

Sensitivity analysis was performed through randomly excluding one trial as well as interchanging the fixed-effects model with the random-effects model from pooled analysis. The outcomes were confirmed to be stable upon sensitivity analysis. Meanwhile, publication bias was assessed using funnel plots. Specifically, complication was treated as an exemplary indicator for publication bias assessment. No distinct asymmetry could be observed from the shape of funnel plot, suggesting no proof of publication bias. (Fig. 6).

## Discussion

It is demonstrated in the current meta-analysis that, obese patients undergoing MI-TLIF have experienced shorter operative time, less intraoperative blood loss, less postoperative drainage, and shorter LOS than those in Open-TLIF group. Moreover, our study also discovers that MI-TLIF can reduce the early BP-VAS score compared with Open-TLIF. However, no differences are founded in ODI, late BP-VAS, early LP-VAS and late LP-VAS scores. Furthermore, MI-TLIF therapy can also evidently decrease the complication rates.

Additionally, this review also suggests marked reduction in operative time and LOS in patients receiving MI-TLIF, which is consistent with systematic reviews [12–14] reporting lumbar disease in general. For instance, Lee et al. [31] and Schizas et al. [32] had indicated markedly decreased operative time accompanied by the increase in number of MI-TLIFs performed. However, several studies have reported a trend of longer operative time for MI-TLIF group [33–35]. Such inconsistency may be ascribed to the fact that MI-TLIF is a more technically demanding procedure in the limited space. In addition, spine surgeons have accumulated their experience with the growingly popular MI procedure, thus resulting in less reported operative time. Moreover, the less blood loss and postoperative drainage may benefit from the less muscle damage in MIS-TLIF than in Open-TLIF. Obese patients undergoing MI-TLIF can initiate the off-bed activity early, which is highlighted by the following reasons. Firstly, there is less spinal muscle atrophy and blood supply disturbances in MI-TLIF than those observed in Open-TLIF. Secondly, smaller incision and less retraction may promote faster recovery, which is particularly applicable for those with hematologic and immune-related conditions who especially benefit from less blood loss and less infection exposure risk. In addition, MI-TLIF therapy has outstandingly reduced the complication rates, which is consistent with the results reported by Khan in 2015 [12]. In fact, the difference in complication rates becomes increasingly pronounced with the increase in obesity [25], which may be mainly related to the decreased infection and lower blood loss [27, 28].

In terms of the functional and pain outcomes, this review demonstrates that MI-TLIF can only reduce the early BP-VAS score when comparing the ODI and VAS measures. In contrast, Goldstein et al. [13] and Tian et al. [14] noted a trend toward more marked improvements in VAS and ODI for MI-TLIF at long-term follow-up. However, normal weight patients were also enrolled in their trails. Similar to our findings, Lu et al. [36] reported no obvious overall difference between MI-TLIF and Open-TLIF in terms of functional and pain outcomes (≥12 mo). Nevertheless, the early VAS and early ODI (≤6mo) were not analyzed in their research. In our study, no more prominent improvement can be observed in early BP-VAS score after MI-TLIF, which is also limited by the low number of studies enrolled. Therefore, we propose that early and late VAS and ODI scores should also be included as standard reported measures of outcomes for future studies defining these important patient-reported variables.

Nonetheless, the current study is inevitably associated with certain limitations. Firstly, all the included studies are observational trials and no RCT is enrolled in this analysis, which is responsible for the low level of evidence for this meta-analysis. Secondly, heterogeneity can be observed in some of the analyses, and efforts have been made to determine the cause using sensitivity analysis. Thirdly, 4 of the 7 studies enrolled in the meta-analysis do not carry out follow-up for a long enough period. Additionally, unpublished studies are not included because of the difficulty in accessing their data, but no evidence of publication bias is observed in the results.

## Conclusion

In conclusion, findings in current study demonstrate that MI-TLIF is associated with shorter operative time, less intraoperative blood loss, less postoperative drainage, fewer complications and shorter LOS in obese patients, despite of the above limitations. MI-TLIF can lower the early BP-VAS score; nevertheless, the long-term functional and pain outcomes are similar between MI-TLIF and Open-TLIF groups. Therefore, large double-blind and randomized-control trials are required to evaluate the safety, efficacy and quality of life in obese patients following lumbar spinal fusion surgery.

## Additional file

Additional file 1: Table S1. The detail of Preferred Reporting Items for Systematic Reviews and Meta-Analyses checklist. (DOC 64 kb)

## Abbreviations

BMI: Body mass index
BP-VAS: Back pain-Visual Analog Scale
CI: Confidence interval
CNKI: China National Knowledge Internet
LOS: Length of stay
MD: Mean difference
MI-TLIF: Minimally invasive transforaminal lumbar interbody fusion
NOS: Newcastle–Ottawa Scale
ODI: Oswestry Disability Index
Open-TLIF: Open transforaminal lumbar interbody fusion surgery
OR: Odds ratio

## References

[1] Deyo RA, Bass JE (1989). Lifestyle and low-back pain. The influence of smoking and obesity. Spine.

[2] Clinical Guidelines on the Identification, Evaluation, and Treatment of Overweight and Obesity in Adults (1998). The evidence report. National Institutes of Health. Obes Res.

[3] Sielatycki JA, Chotai S, Stonko D, Wick J, Kay H, McGirt MJ, Devin CJ (2016). Is obesity associated with worse patient-reported outcomes following lumbar surgery for degenerative conditions?. Eur Spine J.

[4] Dario AB, Ferreira ML, Refshauge K, Sanchez-Romera JF, Luque-Suarez A, Hopper JL, Ordonana JR, Ferreira PH (2016). Are obesity and body fat distribution associated with low back pain in women? A population-based study of 1128 Spanish twins. Eur Spine J.

[5] Vaidya R, Carp J, Bartol S, Ouellette N, Lee S, Sethi A (2009). Lumbar spine fusion in obese and morbidly obese patients. Spine.

[6] Djurasovic M, Bratcher KR, Glassman SD, Dimar JR, Carreon LY (2008). The effect of obesity on clinical outcomes after lumbar fusion. Spine.

[7] Patel N, Bagan B, Vadera S, Maltenfort MG, Deutsch H, Vaccaro AR, Harrop J, Sharan A, Ratliff JK (2007). Obesity and spine surgery: relation to perioperative complications. Journal of neurosurgery Spine.

[8] McGuire KJ, Khaleel MA, Rihn JA, Lurie JD, Zhao W, Weinstein JN (2014). The effect of high obesity on outcomes of treatment for lumbar spinal conditions: subgroup analysis of the spine patient outcomes research trial. Spine.

[9] De la Garza-Ramos R, Bydon M, Abt NB, Sciubba DM, Wolinsky JP, Bydon A, Gokaslan ZL, Rabin B, Witham TF (2015). The impact of obesity on short- and long-term outcomes after lumbar fusion. Spine.

[10] Dhall SS, Wang MY, Mummaneni PV (2008). Clinical and radiographic comparison of mini-open transforaminal lumbar interbody fusion with open transforaminal lumbar interbody fusion in 42 patients with long-term follow-up. J Neurosurg Spine.

[11] Lau D, Lee JG, Han SJ, Lu DC, Chou D (2011). Complications and perioperative factors associated with learning the technique of minimally invasive transforaminal lumbar interbody fusion (TLIF). J Clin Neurosci.

[12] Khan NR, Clark AJ, Lee SL, Venable GT, Rossi NB, Foley KT (2015). Surgical outcomes for minimally invasive vs open Transforaminal lumbar Interbody fusion: an updated systematic review and meta-analysis. Neurosurgery.

[13] Goldstein CL, Macwan K, Sundararajan K, Rampersaud YR (2014). Comparative outcomes of minimally invasive surgery for posterior lumbar fusion: a systematic review. Clin Orthop Relat Res.

[14] Tian NF, Wu YS, Zhang XL, Xu HZ, Chi YL, Mao FM (2013). Minimally invasive versus open transforaminal lumbar interbody fusion: a meta-analysis based on the current evidence. Eur Spine J.

[15] Skovrlj B, Belton P, Zarzour H, Qureshi SA (2015). Perioperative outcomes in minimally invasive lumbar spine surgery: a systematic review. World J Orthod.

[16] Wang XS, Sun RF, Ji Q, Zhao B, Niu XM, Wang R, Peng L, Tian XD (2014). A meta-analysis of interlaminar minimally invasive discectomy compared to conventional microdiscectomy for lumbar disk herniation. Clin Neurol Neurosurg.

[17] Phan K, Rao PJ, Mobbs RJ (2015). Percutaneous versus open pedicle screw fixation for treatment of thoracolumbar fractures: systematic review and meta-analysis of comparative studies. Clin Neurol Neurosurg.

[18] Moher D, Liberati A, Tetzlaff J, Altman DG, Group P (2009). Preferred reporting items for systematic reviews and meta-analyses: the PRISMA statement. PLoS Med.

[19] Flegal KM, Carroll MD, Kuczmarski RJ, Johnson CL (1998). Overweight and obesity in the United States: prevalence and trends, 1960-1994. Int J Obes Relat Metab Disord.

[20] Oremus M, Oremus C, Hall GB, McKinnon MC, Ect, Cognition Systematic Review T (2012). Inter-rater and test-retest reliability of quality assessments by novice student raters using the Jadad and Newcastle-Ottawa scales. BMJ Open.

[21] Deeks JJ (2002). Issues in the selection of a summary statistic for meta-analysis of clinical trials with binary outcomes. Stat Med.

[22] Higgins JP, Thompson SG (2002). Quantifying heterogeneity in a meta-analysis. Stat Med.

[23] Begg CB, Mazumdar M (1994). Operating characteristics of a rank correlation test for publication bias. Biometrics.

[24] Adogwa O, Carr K, Thompson P, Hoang K, Darlington T, Perez E, Fatemi P, Gottfried O, Cheng J, Isaacs RE (2015). A prospective, multi-institutional comparative effectiveness study of lumbar spine surgery in morbidly obese patients: does minimally invasive transforaminal lumbar interbody fusion result in superior outcomes?. World Neurosurg.

[25] Lau D, Khan A, Terman SW, Yee T, La Marca F, Park P (2013). Comparison of perioperative outcomes following open versus minimally invasive transforaminal lumbar interbody fusion in obese patients. Neurosurg Focus.

[26] Mao KY, Zhang Q, Shi T, Su XZ, Xiong S, Wang B, Gu TS, Liu JH, Zhang YB, Han ZC (2015). Therapeutic effect comparison of minimally invasive surgery and open transforaminal lumbar interbody fusion in treatment of obese patients with lumbar intervertebral disc. Acad J Chin Pla Med School.

[27] Terman SW, Yee TJ, Lau D, Khan AA, La Marca F, Park P (2014). Minimally invasive versus open transforaminal lumbar interbody fusion: comparison of clinical outcomes among obese patients. J Neurosurg Spine.

[28] Wang J, Zhou Y, Feng Zhang Z, Qing Li C, Jie Zheng W, Liu J (2014). Comparison of the clinical outcome in overweight or obese patients after minimally invasive versus open transforaminal lumbar interbody fusion. J Spinal Disord Tech.

[29] Wang YP, An JL, Sun YP, Ding WY, Shen Y, Zhang W (2017). Comparison of outcomes between minimally invasive transforaminal lumbar interbody fusion and traditional posterior lumbar intervertebral fusion in obese patients with lumbar disk prolapse. Ther Clin Risk Manag.

[30] Zhang Q, Mao KY, Wang B, Gu TS, Xiong S, Zhang YB, Han ZC, Wang YG, Xiao B (2015). Clinical effects of minimally invasive transforaminal lumbar interbody fusion for obese ;patients with lumbar disc herniation in peri operation period. Int J Orthop.

[31] Lee JC, Jang HD, Shin BJ (2012). Learning curve and clinical outcomes of minimally invasive transforaminal lumbar interbody fusion: our experience in 86 consecutive cases. Spine.

[32] Schizas C, Tzinieris N, Tsiridis E, Kosmopoulos V (2009). Minimally invasive versus open transforaminal lumbar interbody fusion: evaluating initial experience. Int Orthop.

[33] Shunwu F, Xing Z, Fengdong Z, Xiangqian F (2010). Minimally invasive transforaminal lumbar interbody fusion for the treatment of degenerative lumbar diseases. Spine.

[34] Adogwa O, Parker SL, Bydon A, Cheng J, McGirt MJ (2011). Comparative effectiveness of minimally invasive versus open transforaminal lumbar interbody fusion: 2-year assessment of narcotic use, return to work, disability, and quality of life. J Spinal Disord Tech.

[35] Wang J, Zhou Y, Zhang ZF, Li CQ, Zheng WJ, Liu J (2010). Comparison of one-level minimally invasive and open transforaminal lumbar interbody fusion in degenerative and isthmic spondylolisthesis grades 1 and 2. Eur Spine J.

[36] Lu VM, Kerezoudis P, Gilder HE, McCutcheon BA, Phan K, Bydon M (2017). Minimally invasive surgery versus open surgery spinal fusion for Spondylolisthesis: a systematic review and meta-analysis. Spine.

